# Ability of Selected Monoterpenes to Reduce Fe(III) Ions Being Pro-Neurodegenerative Factors: Tests Based on a FRAP Reaction Extended to 48 Hours

**DOI:** 10.3390/ijms25042191

**Published:** 2024-02-12

**Authors:** Karolina A. Wojtunik-Kulesza, Anna Oniszczuk

**Affiliations:** Department of Inorganic Chemistry, Medical University of Lublin, 20-059 Lublin, Poland; anna.oniszczuk@umlub.pl

**Keywords:** FRAP, monoterpene, Tween 20, linalool, isopulegol, terpinene, carvone, citral, phellandrene

## Abstract

Monoterpenes are secondary plant metabolites, and such volatile compounds have antioxidant, antibacterial, antiviral, and enzyme inhibitory properties. These compounds are also able to reduce the potentially pro-neurodegenerative trace metal ions that can be sources of free radicals. One basic method used to evaluate the ability of chemical compounds to reduce Fe(III) is FRAP. To date, most studies based on a FRAP assay were performed within several dozen minutes. However, taking into account the diversity of compounds, it is justified to observe their activity over a much longer period of time. The present study aimed to observe the activity of isopulegol, γ-terpinene, α-terpinene, linalool, carvone, citral, and α-phellandrene over a 48 h period. Our study indicates that the lengthened reaction period enhanced activity from several dozen to several hundred percent. The obtained results also revealed an explicit high correlation of the increase in the activity of compounds with the increase in monoterpene concentration. Due to the hydrophobic character of monoterpenes, the FRAP method was modified by the addition of Tween 20. The highest activity was obtained for α-terpinene and γ-terpinene.

## 1. Introduction

Terpenes, a group of isoprene derivatives, are a highly diversified category of secondary plant metabolites. Among them are the monoterpenes. These are two linked isoprene units [1], and the compounds are responsible for the aroma of plants including herbs commonly used in daily life. Due to their intense smell, monoterpenes are widely used in many industrial fields such as spices, cosmetics, flavors, etc. Nevertheless, most studies are based on their biological activities such as anticancer, antibacterial, and antifungal that reveal cardiovascular protective properties and are used in pharmacology and medicine [2]. The secondary plant metabolites are often considered as active anti-neurodegenerative natural compounds which are able to counteract a few neurodegenerative factors [3]. It is known that these compounds can act as free radical scavengers, antioxidants, AChE inhibitors, metal ion reductors, and a lot more. The great interest in these compounds results from the fact that they are low-molecular-weight compounds of a lipophilic nature [4]. The group of monoterpenes include acyclic (i.e., geraniol, myrcene), monocyclic (i.e., thymol, menthol) and bicyclic compounds (i.e., pinene, myrtenal). These are found in the form of alcohols, ketones, aldehydes, etc. [1]. The diversified chemical structures modified by various moieties bring the aforementioned biological activity [4,5,6].

Although they are a large and diversified group of secondary plant metabolites, the compounds are not as well characterized as other groups, i.e., phenols. There are a limited number of studies, both in vitro and in vivo, based on the specific compounds. The base of most of these are essential oils rich in terpenes and terpenoids. Considering the activity of the selected monoterpenes on their anti-neurodegenerative properties, a significant part of the analysis is based on the multifactorial character of the compounds [7]. It is known that most are able to counteract oxidative changes, and selected monoterpenes can inhibit acetylcholinesterase, as well as reduce Cu(II) and Fe(III) [5,8]. The latter activity is of particular interest recently. This phenomenon results from fact that too high levels of trace metal ions explicitly induce neurodegenerative changes. One of possible way to counteract the effect is chelation or reduction of Fe(III) ions. 

A method most often used for the in vitro evaluation of ferric ion reduction is FRAP (ferric reducing antioxidant power). The colorimetric assay is based on the ability of antioxidants/reducers to reduce a colorless complex of 2,4,6-tripyridyl-s-triazine-Fe^3+^ (TPTZ-Fe^3+^) to an intensive blue TPTZ-Fe^2+^ complex. A characteristic feature of FRAP, unlike other single electron transfer (SET) -based methods, is the requirement of the presence of an acidic pH (pH = 3.6) so as to engender iron solubility [9]. The low pH leads to a decrease in the ionization potential that drives electron transfer and an increase in the redox potential that causes a shift in the dominant reaction mechanism. It is worth mentioning that, despite the aforementioned drawbacks, the method is cheap, simple, relatively rapid, and does not require specialized equipment. 

The original methodology, presented by Benzie and Strain, recommends leaving the sample for 4 min, as this they considered to be a sufficient time for the reduction reaction to occur [10]. Detailed long-lasting studies have revealed, however, that the time can be insufficient. Pulido, Bravo, and Saura-Calixto, for example, showed that the reduction potential can be observed within a few minutes to several hours [11]. This comes about because various types of antioxidants can require different times for the specific reaction to complete. Therefore, a single-point absorption endpoint can be insufficient for the reaction, and disparate reaction times have to be provided. 

Overall, FRAP is considered to be the most useful SET-based method that is not based on radical scavenging (hydrogen atom transfer (HAT) mechanism). Hence, the results cannot be correlated with other antioxidant activity measurements. Nevertheless, obtained outcomes can be considered along with other results that employ different mechanisms of action [12]. A significant limitation of the method is the need for an aqueous environment of reaction, hence lipophilic compounds are difficult to examine. In this case, method modification is needed. 

The aim of the presented paper was to observe the kinetic mechanism of Fe(III) reduction by selected monoterpenes within 48 h. For monoterpenes, this is the first study of this type of reaction kinetics using a long-lasting FRAP assay. The first part of the study [5] revealed satisfactory activity of the compounds towards the reduction of ferric ions within 20 min. The seven most active compounds, as found in this work, were reinvestigated in the present study. Due to the fact that various compounds have different reaction times, it was decided to observe the reaction for 48 h. This approach ensured the analysis of the activity of monoterpenes with a slow ion reduction mechanism. Moreover, the research will expand knowledge about the anti-neurodegenerative activity of monoterpenes. We know that Fe(III) ions contribute to the development of dementia, including Alzheimer’s disease [13]. Studying the reaction kinetics of monoterpenes will allow for a more accurate characterization of the potential multidirectional effects of these compounds on neurodegeneration.

## 2. Results

**Figure 1:**
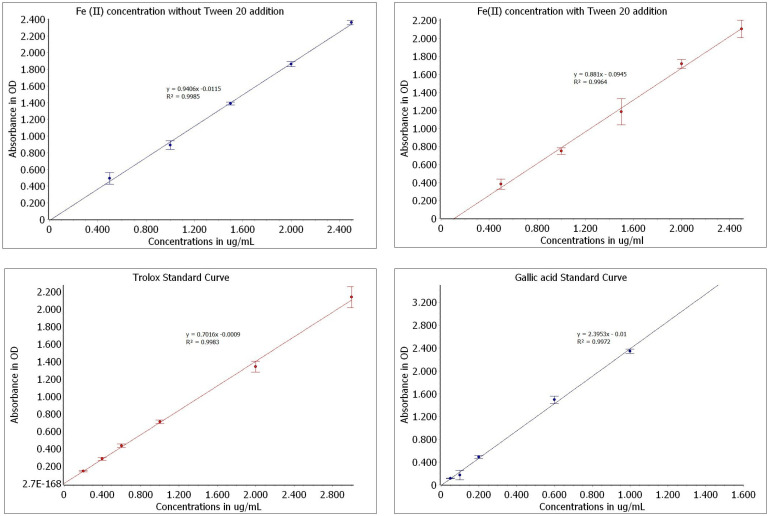
Standard curves used for calculation of monoterpene reduction activity.

The activity of each of the seven studied monoterpenes was evaluated under two different conditions: (1) a normal basic solution of FRAP and (2) a solution enriched with Tween 20. The obtained results were calculated based on standard curves (Figure 1) for Fe(II) concentration, gallic acid, and trolox. 

**Figure 2:**
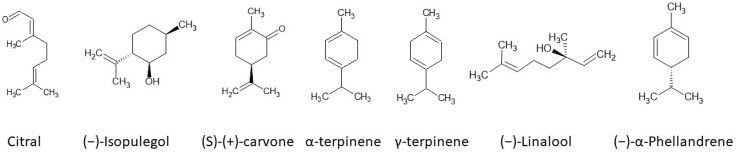
Monoterpenes studied towards Fe(III) reduction activity.

Spectrophotometric analysis of the selected monoterpene’s (Figure 2) ability to reduce Fe(III) ions was evaluated by applying the FRAP method. The following concentrations were used: 100 μM, 250 μM, 500 μM, 750 μM, 1 mM, 10 mM, and 100 mM. It is known that, from pharmaceutical and medical points of view, the most significant and interesting results are those obtained for the lowest concentrations. However, considering the kinetics of the reactions, as well as the general characteristics of monoterpene reduction activity, high concentrations of the compounds can also be analyzed. 

### 2.1. General Reduction Activity of Monoterpenes

In analyzing the activity of each compound within 48 h of the reaction, interesting changes were observed. A detailed analysis of the results obtained for the lowest compound concentration (100 µM) revealed that all were able to reduce Fe(III) (Table 1). However, all values used for activity calculation, namely Fe(II) concentration, gallic acid, and trolox equivalents, changed over time for all the tested monoterpenes. 

Considering the activity of the secondary plant metabolites at a concentration of 100 μM, interesting dependencies were noticed. The most active monoterpenes turned out to be α-terpinene, γ-terpinene, citral, and α-phellandrene. The compounds revealed the highest activity assessed in terms of three values, namely Fe(II) concentration, gallic acid, and trolox equivalents. α-Terpinene and γ-terpinene revealed much better activity than the others due to a higher amount of Fe(II), gallic acid, and trolox equivalents, which were observed during the entire 48-h reaction. Only citral and α-phellandrene revealed slightly better activity at some points during the whole 48-h reaction (Table 1). Lower activity was observed for isopulegol, carvone and linalool (Table 1).

### 2.2. Percentage Analysis of Changes in the Amount of Fe(II) during the Entire 48-H Experiment

The extended time of the monoterpene– Fe(III) reaction had significant influence on the amount of Fe(II) obtained. The number of ferrous ions were observed in the form of an increase in absorbance, reflecting an increase in the production of Fe(II) ions. In some cases, a decrease in absorbance was also observed.

A detailed analysis of the results obtained for the method without Tween 20 modification revealed that almost all monoterpenes revealed enhanced Fe(II) content after 12 h, 24 h, and 48 h of reaction, in comparison to 1 h of reaction (Table 2). This trend was observed for all used concentrations of monoterpene except linalool (250 µM–100 mM for 24 h and 48 h of reaction), α-phellandrene (10 mM and 100 mM for 12 h, 24 h and 48 h for reaction) and α-terpinene (100 mM for 12 h of reaction).

The highest increase was observed for γ-terpinene, for which the increase was above 200% for concentrations of 100 µM, 250 µM, and 500 µM (except 250 µM for 48 h of reaction) and α-phellandrene with an increase above 100% for 12 h, 24 h and 48 h of reaction for concentrations of 100 µM, 250 µM, and 500 µM (Table 2). 

The amount of Fe(II) was unstable, and absorbance-reflecting ion content varied throughout the whole 48 h experiment. Indeed, in certain monoterpenes, the amount of Fe(II) both increased and decreased throughout the whole experiment. For example, linalool revealed enhanced Fe(II) for lower concentrations (100% increasing after 12 h of reaction in comparison to 1 h of reaction for a concentration 100 µM) and a decrease in activity during the next reaction steps (Table 2). Similar changes were observed for citral.

The addition of surfactant to the experiment had influence upon the stability of the reaction, but, simultaneously, various impacts on Fe(II) creation was observed (Table 1). The positive influence was noticed for most of the analyzed monoterpenes, in terms of Fe(II) creation during the whole experiment (Table 1), as well as gallic acid and trolox equivalents. Simultaneously, in some cases (γ-terpinene, isopulegol, and citral) a decrease in the amount of Fe(II) ions (μg/mL) was observed. Additionally, Tween 20 caused a reduction in the increase (in %) in the amount of Fe(II) ions produced within 12 h, 24 h, and 48 h in relation to 1 h of reaction (Table 2). 

### 2.3. Stability of Fe(III) Reduction Reaction and Influence of Tween 20

The 48 h kinetic reaction of Fe(III) reduction allowed for observing the process and evaluating the stability of it, as well as the time–reduction capacity relationship. 

**Figure 3:**
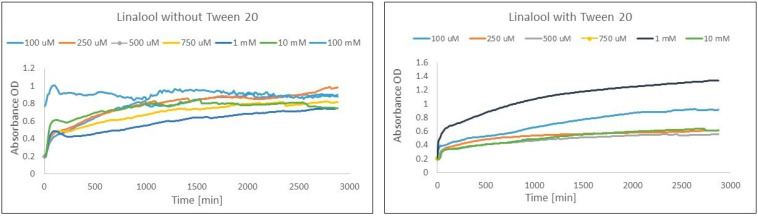
Comparison of the course of the linalool–Fe(III) reaction for the FRAP method before and after adding the surfactant. Diagram with Tween 20 did not include linalool at concentrations of 100 mM due to absorbance showing a value above the detection range.

Considering the influence of Tween 20 on the FRAP experiment, the most explicit positive influence of surfactant was observed for linalool, α-phellandrene, and carvone. An unstable reaction based on the method without surfactant modification was recorded for linalool. This terpene showed significant differences in absorbance during the course of the reaction, and both an increase and a decrease in absorbance were observed during the reaction for all terpene concentrations (Figure 3). In the case of the monoterpenes, the positive impact was observed in terms of a more stable reaction as well as an increase in the amount of Fe(II) during whole reaction in comparison to 1 h of reaction, whereas in the experiment without surfactant, a decrease was observed. 

When Tween 20 was added to the mixture to boost the solubility of the monoterpenes, a simultaneous effect was the improvement in the activity of selected monoterpenes resulting from the elimination of the negative impact of their low solubility in aqueous solutions. Hence, the stability of the reaction was enhanced by the addition of Tween 20. For example, surfactant addition caused a FRAP reaction stabilization for selected concentrations of linalool (Figure 3). Interestingly, the compound revealed a decrease in Fe(II) creation for all concentrations for 24 h and 48 h of reaction in comparison to Fe(II) creation at 1 h of reaction, whereas the addition of a surfactant caused significant increases in Fe(III) reduction activity (from 77% decreasing in reaction without Tween 20 to 105% increasing in activity obtained for a method modified with Tween 20 for the same concentration and reaction time) during the whole reaction in comparison to 1 h of reaction.

**Figure 4:**
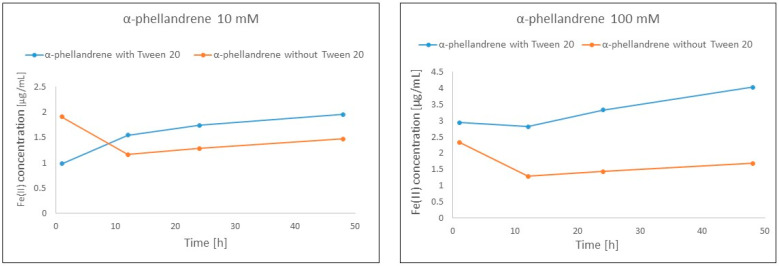
Positive influence of Tween 20 addition on Fe(III) reduction activity of α-phellandrene.

In the case of α-phellandrene, an explicitly positive impact was observed for the highest measured concentrations, namely 10 mM and 100 mM. The results indicate a positive impact on both Fe(II) concentration as well as a reduction of the effect of reducing the number of ions with the progress of the reaction compared to 1 h of reaction (Figure 4).

Tween 20 had a positive impact on the reaction by creating higher stability (in comparison to reactions without surfactant) and also had positive impacts on the absorbance values obtained from different reaction times. The influence can be observed based on all values, namely Fe(II) concentrations, gallic acid, and trolox equivalents. Quantitative comparison allowed us to confirm a positive effect of the surfactant addition, with a few exceptions (Table 1).

### 2.4. Correlation Coefficients for Fe(II) Concentration, Gallic Acid, and Trolox Equivalents

Correlation coefficients are an important element of the quantity analysis of activity. In this study, analysis was performed for all monoterpenes with regard to time of reaction, concentrations, and obtained results for Fe(II) concentration, gallic acid, and trolox equivalents. In a significant number of cases, a very high correlation (above 0.9) of the increase in the activity of compounds with the increase in the concentration of monoterpenes was demonstrated for reactions at 1 h, 12 h, 24 h and 48 h. The highest concentration activity dependency was observed for citral (all correlation coefficients equal to 1.00 for all three activity factors for the method with Tween 20 addition) and γ-terpinene (correlation coefficients equal to 1.00 prevailed for all measurements). 

However, while correlations were observed for all three parameters indicating compound activity, in the case of carvone, isopulegol, and linalool, a weak correlation for some reaction times was observed, and correlation coefficients did not exceed 0.5, and negative values were obtained. Linalool revealed the weakest correlations among all monoterpenes. Herein, in a few cases (gallic acid equivalents obtained for both methods; Fe(II) concentration for 1 h and 12 h for reaction with Tween 20 addition; trolox equivalents for 1 h and 12 h for reaction with Tween 20 addition), a high negative correlation was observed (Table 3).

## 3. Discussion

It is known that reaction kinetics are tightly connected with the structure and the properties of compounds as a whole. In accordance with Pulido et al. [11], determining the activity of compounds within a few minutes of reaction is insufficient. As the authors underlined, full analysis of the activity often requires time periods more extensive than that indicated by the authors of the FRAP method [10]. Hence, in order to properly evaluate the activity of selected compounds, the reaction should be performed for time periods sometimes ranging from several dozen to several hundred minutes or even longer.

To date, available data does not reveal the activity of monoterpenes towards Fe(III) reduction within a long time period. Moreover, no published study has revealed the kinetics of the reaction. A basic approach to mitigate this lack of information was presented in a previous paper. In this, selected monoterpenes were studied for Fe(III) reduction, along with a modification of FRAP method by the addition of Tween 20 [5]. The results obtained revealed the positive influence of surfactant on monoterpene activity. However, the studies were performed within a basic reaction time of 20 min. In order to evaluate the activity in more detail, in the current paper, the activity of the most promising monoterpenes were evaluated within a 48 h period. 

The obtained results explicitly indicate the activity of selected monoterpenes in reducing Fe(III) within 48 h. Our findings indicate that there are compounds in which activity increased as the reaction time increased (γ-terpinene, carvone, and isopulegol), or the activity was relatively stable (citral), or the activity changed enough to reach negative absorbance at various stages of reaction (linalool and α-phellandrene for various concentrations and time of reaction). The unstable reaction effect is interesting and simultaneously hard to interpret. It is known that monoterpenes are hydrophobic compounds, and the negative effect was minimized by dissolving the compounds in methanol in order to obtain specific concentrations of the solution, as well as to improve their solubility. The logP parameter is important for considerations of compound solubility. The value allows for comparing the solubility of various compounds and simultaneously enables interpreting differences in activity related with the solubility of it. Herein, negative values indicate more hydrophilic compounds (logP = 0 is characteristic for compounds equally partitioned between lipid and aqueous phases), and positive values indicate a hydrophobic character [12]. Table 4 presents logP values for the selected studied monoterpenes.

As revealed in Table 4, all the analyzed monoterpenes demonstrate hydrophobic character, thus their solubility in the FRAP solution is very weak. Isopulegol holds the lowest hydrophobic character, whereas α- and γ-terpinene display the highest. The low solubility of the compounds was enhanced by dissolving the samples in methanol before being added into the monoterpene–FRAP solution mixture. The analysis did not demonstrate cloudiness or a resulting two-phase solution; rather, the differences in solubility were revealed as an unstable character of reaction that was improved by the addition of Tween 20. The change could be easily observed for α-phellandrene wherein the reaction was more stable after the addition of a surfactant, and the compound demonstrated a higher absorbance due to higher concentrations of Fe(II) (Figure 4). 

Correlation coefficient values are also important for results interpretation. In our study, the calculations were performed for monoterpene concentrations, including the level of Fe(II), gallic acid equivalents, and trolox equivalents. In the majority of cases, high correlation (>0.9) was obtained. In contrast, linalool and isopulegol demonstrated the lowest correlation (Table 3). Here, linalool revealed a high negative correlation for the monoterpene concentration (Fe(II) concentration for the assay modified by the addition of Tween 20), as well as for monoterpene concentration (trolox equivalents for the assay with surfactant modification and for gallic equivalents for both methods). A negative correlation describes a situation where one variable increases in value while the other decreases [14]. This means that, for example, when the concentration of linalool increases, its Fe(II) concentration or trolox equivalents decreases. The situation is the opposite of a positive correlation, where both variables increase or decrease at the same time. This effect can be observed in the remaining monoterpenes. In the case of linalool, there is a negative correlation (−0.29 and −0.30) for Fe(II) with monoterpene concentrations for the method modified by the addition of Tween 20, as obtained after 1 h and 12 h of reaction. The values explicitly indicate that linalool is able to reduce Fe(III) ions within a long period of time, whereas effectiveness is the highest for low concentrations at the first stage of reaction, and the positive impact of higher concentrations (positive correlation coefficients 0.97 and 0.87) is observed in later stages of reaction. This phenomenon is crucial for considering linalool as an iron ion reducer.

It is known that high absorbance indicates high activity of compounds towards Fe(III) reduction and to the creation of dark blue complex TPTZ-Fe(II) ions. Thus, higher levels of Fe(II) create a dark solution and, thus, high absorbance. In the case of lower absorbance, there can be a phenomenon based on oxidation of Fe(II) to Fe(III) that leads to solution discoloration. This effect can result from the too weak character of the studied antioxidants to maintain iron in the (II) oxidation state and may result in re-oxidation of Fe(II) to Fe(III). This phenomenon must be analyzed in more detail. 

Studies based on monoterpene–Fe(III) reaction were performed several times in various reaction conditions. Among these is an in vitro FRAP assay performed for α-phellandrene [15]. Results revealed FRAP IC50 for the compound equal to 1619.6 µM, whereas gallic acid IC50 was equal to 123.1 µM. Interesting results were also obtained by Safaeian et al. who decided to determine intra- and extra-cellular FRAP values for citral using a commercial kit [16]. The analysis was performed for 40 min, and the results revealed that pretreatment with citral significantly increased the FRAP value in intracellular fluids at concentrations of 1.25, 2.5, 5, and 10 µg/mL. Indeed, the authors saw dose- and time-dependent citral activity towards the protection of normal cells against distressing stimuli. 

Similar studies based on an original FRAP assay confirmed the ability of citral to reduce Fe(III) [17]. In their work, the highest concentration of the terpene (300 µg/mL) revealed a satisfactory ability to reduce Fe(III). Nevertheless, the assays were performed at standard times of 20 min or 40 min, which can impact the actual total activity of the compound. Confirmation of this fact is found in Henriques et al., who observed that FRAP values obtained for apple and berry extracts depended on incubation time [18]. The results revealed that the standard 4 min FRAP assay method does not reflect the total antioxidant capacity.

Significant studies based on FRAP assays were also performed for phenols. The results confirm the theory that the reaction time of the standard FRAP procedure is too short and does not reflect the actual activity of the compounds [19]. Results demonstrated that absorbance obtained at 48 h of reaction was higher by 5.395% for vanillin, 426% for caffeic acid, 170% for sinapinic acid, 67% for gallic acid, 19% for syringic acid, and only by 4% for Trolox, in comparison to values obtained at 10 min of reaction. This value requires longer periods of incubation time. Because of this, a kinetic FRAP profile should be realized for each type of sample analyzed in order to establish the most suitable incubation period. 

In our current study, all seven studied monoterpenes revealed activity to reduce Fe(III). Among these, α-terpinene, γ-terpinene, citral, and α-phellandrene showed the highest activity. In the study, activity was observed based on three values, namely Fe(II) concentration, gallic acid, and trolox equivalents. It is important to underline the negative correlation coefficients for linalool, as this clearly indicates that lower concentrations of the monoterpenes are more effective for ferric ion reduction abilities for selected stages of reaction.

In considering the activity of the terpenes as potential antioxidants, ADMET analysis should always be applied, as this allows for predicting absorption, distribution, metabolism, elimination, and the toxicity of selected compounds. In the active monoterpenes, the analysis indicates that the compounds correctly obeyed the Lipinski Rule of Five, including high intestinal absorption and crossing the blood–brain barrier with ease, which is a huge advantage, taking into account high levels of Fe(III) and their harmful activity in the brain.

## 4. Materials and Methods

### 4.1. Materials

The following terpenes were obtained from Sigma-Aldrich: (−)-isopulegol (≥99%), γ-terpinene (97%), α-terpinene (≥95%), (−)-linalool (≥97%), (S)-(+)-carvone (≥96%), citral (≥95%) (mixture of isomers), (−)-α-phellandrene (≥90%) and TPTZ (2,4,6-Tri(2-pyridyl)-s-triazine)). Acetic acid (ACS), FeCl_3_ × 6H_2_O, FeSO_4_ × 7H_2_O, phosphoric acid (ACS), hydrochloric acid (ACS), and metanol (analytic purity grade) were obtained from Polish Reagents (Gliwice, Poland). The standards trolox ((±)-6-hydroxy-2,5,7,8-tetramethylchromane-2-carboxylic acid, 97%), gallic acid (>98%), and the surfactant Tween 20 were purchased from Sigma-Aldrich.

### 4.2. Methods

The FRAP assay was based on [5] with some modifications. The FRAP solution was prepared fresh each time by mixing (10:1:1 *v*/*v*/*v*) 0.3 M acetate buffer (pH 3.6), 0.01 M TPTZ in 0.04 M HCl, and 0.02 M FeCl_3_ × 6H_2_O and kept in dark. Terpenes were dissolved in methanol in order to provide specific final concentrations. An appropriate amount of studied terpene was mixed with 200 μL of the FRAP solution in a microplate, and the monoterpene activity was evaluated for the following concentrations: 100 μM, 250 μM, 500 μM, 750 μM, 1 mM, 10 mM, and 100 mM. The prepared samples were vortexed and incubated at 37 °C away from light. Thereafter, absorbance was measured at 593 nm using a SPECTROstar Nano microplate reader at regular intervals up to 48 h. The FRAP working solution with methanol instead of a sample was used as a blank. The results were calculated into µg/mL Fe^2+^ on the calibration curve which was prepared analogically using an aqueous solution of FeSO_4_ at concentrations of 0.5–2.5 µg/mL (y = 0.9406x − 0.0115, R² = 0.9964). 

In order to evaluate the impact of surfactant on the Fe(III) reduction activity and solubility of the monoterpenes, in the second experiment, Tween 20 was added to the FRAP solution. Critical Micelle Concentration (CMC) was not exceeded, which, for Tween 20, is equal 0.06 mM. For this purpose, 3 µL of surfactant was added to a mixture solution. All procedures of measurement were identical with the first part of the study. A calculation curve for Fe^2+^ was then prepared (y = 0.881x − 0.0945; R² = 0.9964).

In both cases, absorbance was recorded every 15 min for 48 h. Taking into account the high volatility of terpenes and the long duration of experiments, the plates were protected with a special foil for microplates, SealPlate^®^. The obtained results for each monoterpene are the average of absorbance obtained for four wells.

Additionally, trolox and gallic acid were used as standards. Calibration curves were prepared analogically using methanolic (for trolox) and aqueous (for gallic acid) solutions. The concentrations were selected based on the obtained absorbance results of the tested terpenes to ensure an appropriate range of absorbance values. The following concentrations were used: trolox [0.2–3.0 µg/mL], gallic acid [0.05–1.0 µg/mL]. The following curve parameters were obtained: trolox, y = 0.7016x − 0.0009; R² = 0.9983; gallic acid, y = 2.3953x − 0.01; R² = 0.9972 (Figure 1).

### 4.3. Statistical Analysis

Statistical analysis was performed using Mars, the statistical software built into the microplate reader. The statistical significance of the means was assessed using a one-way analysis of variance (ANOVA). Correlation coefficients were also evaluated.

## 5. Conclusions

Monoterpenes are secondary plant metabolites revealing various biological activities. In order to evaluate their actual activity towards Fe(III) reduction, in this study, the FRAP assay has been modified by the addition of Tween 20, and the reaction time was extended to 48 h. The obtained results explicitly indicate the ability of the selected monoterpenes to reduce ferric ions and, simultaneously, that their hydrophobic nature did not have a terminating effect on the course of the reaction. Among the analyzed compounds, the most effective were α-terpinene and γ-terpinene, followed by citral and α-phellandrene, but the remaining terpenes also revealed good activity. Long periods of reaction caused increases in their activity from several dozen to several hundred percent, i.e., 282% increase for γ-terpinene at 12 h of reaction time and 269% at 48 h of reaction time, in comparison to 1 h of reaction for a monoterpene concentration of 100 µM. The addition of Tween 20 brought about a higher stability within the entire 48 h of reaction for linalool and α-phellandrene. The influence must, however, be considered individually for each monoterpene. Positive impact of surfactant addition was observed for almost all monoterpenes in terms of amount of Fe(II) created during whole reaction. It is interesting to note the fact that Tween 20 caused a reduction in the increase (in %) in the amount of Fe(II) ions produced in relation to 1 h of the reaction. The activity of the selected monoterpenes can be considered as a promising anti-neurodegenerative factor due to the reduction of Fe(III) to Fe(II) and can counteract a few neurodegenerative risk factors.

## Figures and Tables

**Table 1:** Fe (II) concentrations, gallic acid and trolox equivalents obtained for monoterpenes at a concentration of 100 µM studied for Fe(III) reduction activity (n = 4, ±SD). The highest activity is highlighted.

**Monoterpene**	**Fe(II) Concentrations [µg/mL]**							
	**Without Tween 20 Modification**				**With Tween 20 Modification**			
	**1 h**	**12 h**	**24 h**	**48 h**	**1 h**	**12 h**	**24 h**	**48 h**
**α-terpinene**	0.724 ± 0.03	1.39 ± 0.05	1.273 ± 0.05	1.467 ± 0.07	0.835 ± 0.03	1.461 ± 0.06	1.489 ± 0.08	1.545 ± 0.08
**γ-terpinene**	0.356 ± 0.01	1.404 ± 0.08	1.343 ± 0.04	1.355 ± 0.07	0.362 ± 0.02	1.388 ± 0.04	1.394 ± 0.07	1.01 ± 0.04
**isopulegol**	0.49 ± 0.02	0.918 ± 0.03	1.177 ± 0.06	1.311 ± 0.06	0.457 ± 0.02	0.693 ± 0.01	0.825 ± 0.03	1.015 ± 0.05
**citral**	0.683 ± 0.02	1.233 ± 0.04	1.244 ± 0.07	1.606 ± 0.09	0.703 ± 0.04	1.136 ± 0.07	1.247 ± 0.05	1.149 ± 0.06
**α-phellanderene**	0.547 ± 0.01	1.238 ± 0.06	1.367 ± 0.05	1.109 ± 0.05	0.6 ± 0.03	1.281 ± 0.05	1.303 ± 0.06	1.32 ± 0.05
**linalool**	0.527 ± 0.03	1.07 ± 0.04	0.687 ± 0.02	0.819 ± 0.03	0.56 ± 0.02	0.817 ± 0.03	1.085 ± 0.04	1.305 ± 0.07
**carvone**	0.46 ± 0.03	0.911 ± 0.04	1.166 ± 0.05	1.204 ± 0.05	0.566 ± 0.02	0.959 ± 0.05	1.097 ± 0.05	1.246 ± 0.07
**Monoterpene**	**Gallic Acid Equivalents [µg/mL]**							
	**Without Tween 20 Modification**				**With Tween 20 Modification**			
	**1 h**	**12 h**	**24 h**	**48 h**	**1 h**	**12 h**	**24 h**	**48 h**
**α-terpinene**	0.216 ± 0.01	0.411 ± 0.02	0.377 ± 0.02	0.433 ± 0.02	0.248 ± 0.01	0.432 ± 0.02	0.44 ± 0.02	0.456 ± 0.03
**γ-terpinene**	0.108 ± 0.006	0.415 ± 0.03	0.397 ± 0.02	0.401 ± 0.02	0.11 ± 0.006	0.41 ± 0.02	0.412 ± 0.02	0.505 ± 0.03
**isopulegol**	0.147 ± 0.008	0.273 ± 0.01	0.349 ± 0.02	0.388 ± 0.02	0.138 ± 0.007	0.207 ± 0.01	0.245 ± 0.01	0.301 ± 0.02
**citral**	0.204 ± 0.008	0.365 ± 0.02	0.368 ± 0.01	0.474 ± 0.03	0.21 ± 0.01	0.336 ± 0.02	0.369 ± 0.02	0.34 ± 0.02
**α-phellanderene**	0.164 ± 0.008	0.367 ± 0.01	0.404 ± 0.03	0.329 ± 0.01	0.18 ± 0.009	0.379 ± 0.02	0.385 ± 0.03	0.39 ± 0.01
**linalool**	0.158 ± 0.007	0.317 ± 0.02	0.205 ± 0.02	0.244 ± 0.01	0.168 ± 0.008	0.243 ± 0.01	0.321 ± 0.01	0.386 ± 0.02
**carvone**	0.139 ± 0.005	0.271 ± 0.03	0.345 ± 0.02	0.357 ± 0.02	0.169 ± 0.008	0.285 ± 0.01	0.325 ± 0.03	0.369 ± 0.02
**Monoterpene**	**Trolox Equivalents [µg/mL]**							
	**Without Tween 20 Modification**				**With Tween 20 Modification**			
	**1 h**	**12 h**	**24 h**	**48 h**	**1 h**	**12 h**	**24 h**	**48 h**
**α-terpinene**	0.724 ± 0.03	1.39 ± 0.07	1.273 ± 0.06	1.467 ± 0.07	0.835 ± 0.05	1.461 ± 0.08	1.489 ± 0.07	1.545 ± 0.09
**γ-terpinene**	0.356 ± 0.02	1.404 ± 0.07	1.343 ± 0.07	1.355 ± 0.07	0.362 ± 0.02	1.388 ± 0.09	1.394 ± 0.06	1.71 ± 0.05
**isopulegol**	0.49 ± 0.02	0.918 ± 0.05	1.177 ± 0.07	1.311 ± 0.06	0.457 ± 0.03	0.693 ± 0.05	0.825 ± 0.05	1.015 ± 0.08
**citral**	0.683 ± 0.03	1.233 ± 0.06	1.244 ± 0.06	1.606 ± 0.04	0.703 ± 0.06	1.136 ± 0.03	1.247 ± 0.09	1.149 ± 0.06
**α-phellanderene**	0.547 ± 0.03	1.238 ± 0.06	1.367 ± 0.08	1.109 ± 0.03	0.6 ± 0.06	1.281 ± 0.06	1.303 ± 0.08	1.32 ± 0.07
**linalool**	0.527 ± 0.02	1.07 ± 0.07	0.687 ± 0.05	0.819 ± 0.02	0.56 ± 0.03	0.817 ± 0.04	1.085 ± 0.08	1.305 ± 0.06
**carvone**	0.46 ± 0.02	0.911 ± 0.05	1.166 ± 0.05	1.204 ± 0.08	0.566 ± 0.02	0.959 ± 0.08	1.097 ± 0.05	1.246 ± 0.05

**Table 2:** Increase or decrease (%) in Fe(II) content compared to 1 h of reaction. Please see color indications: increasing < 10%, increasing 10–99%, increasing 100–199%, increasing > 200%, decreasing.

Monoterpene	Monoterpene Concentration	Fe(II) Increase or Decrease (%) in Comparison to 1 h of Reaction					
		Without Tween 20 Addition			Assay with Tween 20 Addition		
		12 h	24 h	48 h	12 h	24 h	48 h
**α-terpinene**	100 µM	87%	72%	97%	65%	2%	73%
	250 µM	94%	85%	89%	86%	8%	121%
	500 µM	108%	106%	99%	74%	2%	110%
**γ-terpinene**	100 µM	282%	266%	269%	208%	209%	273%
	250 µM	354%	249%	165%	193%	233%	170%
	500 µM	315%	314%	331%	215%	218%	231%
**isopulegol**	100 µM	85%	136%	162%	40%	62%	94%
	250 µM	60%	112%	117%	67%	83%	82%
	500 µM	45%	72%	86%	39%	54%	81%
**citral**	100 µM	79%	81%	133%	52%	65%	53%
	250 µM	95%	91%	80%	58%	55%	48%
	500 µM	89%	97%	67%	61%	64%	70%
**α-phellandrene**	100 µM	123%	146%	100%	93%	96%	98%
	250 µM	110%	129%	117%	79%	91%	71%
	500 µM	113%	132%	114%	83%	99%	105%
**linalool**	100 µM	100%	29%	54%	37%	76%	108%
	250 µM	76%	−47%	−12%	39%	49%	62%
	500 µM	65%	−50%	−29%	22%	40%	51%
**carvone**	100 µM	95%	148%	156%	56%	76%	97%
	250 µM	95%	113%	148%	48%	60%	84%
	500 µM	66%	99%	126%	30%	42%	51%

**Table 3:** Correlation coefficients for all monoterpene concentrations and (1) Fe(II) concentration, (2) gallic acid equivalents, and (3) trolox equivalents obtained. Please see color indications for correlation coefficients: ≤0.5, 0.51–0.84, 0.85–1.0 along with their shades depending on the detailed values.

**Monoterpene**	**Correlation Coefficients for Fe(II) Concentration**							
	**Without Tween 20 Modification**				**With Tween 20 Modification**			
	**1 h**	**12 h**	**24 h**	**48 h**	**1 h**	**12 h**	**24 h**	**48 h**
**α-terpinene**	0.74	0.45	0.59	0.74	1.00	1.00	0.82	0.75
**γ-terpinene**	0.98	0.99	0.96	0.94	0.81	1.00	1.00	0.98
**isopulegol**	0.65	0.39	0.25	0.53	−0.21	0.99	0.98	0.98
**citral**	0.89	0.93	0.98	0.98	1.00	1.00	1.00	1.00
**α-phellanderene**	0.81	0.87	0.86	0.82	0.99	0.99	0.99	0.98
**linalool**	0.98	0.65	0.10	0.03	−0.29	−0.30	0.97	0.87
**carvone**	0.99	0.96	0.99	1.00	−0.29	−0.30	0.97	0.87
**Monoterpene**	**Correlation Coefficients for Gallic Acid Equivalents**							
	**Without Tween 20 Modification**				**With Tween 20 Modification**			
	**1 h**	**12 h**	**24 h**	**48 h**	**1 h**	**12 h**	**24 h**	**48 h**
**α-terpinene**	0.99	0.99	0.99	0.98	1.00	0.82	0.75	0.70
**γ-terpinene**	1.00	0.99	0.96	0.94	1.00	1.00	1.00	0.98
**isopulegol**	0.98	0.40	0.25	0.53	−0.21	0.99	0.98	0.98
**citral**	1.00	0.93	0.98	0.98	1.00	1.00	1.00	1.00
**α-phellanderene**	1.00	0.87	0.86	0.82	0.99	0.99	0.99	0.98
**linalool**	0.42	−0.99	−0.68	−0.73	−0.29	−0.31	0.97	0.87
**carvone**	0.92	0.96	0.99	1.00	0.79	0.94	0.97	0.98
**Monoterpene**	**Correlation Coefficients for Trolox Equivalents**							
	**Without Tween 20 Modification**				**With Tween 20 Modification**			
	**1 h**	**12 h**	**24 h**	**48 h**	**1 h**	**12 h**	**24 h**	**48 h**
**α-terpinene**	1.00	0.45	0.59	0.74	0.97	0.83	0.76	0.70
**γ-terpinene**	1.00	0.99	0.96	0.94	0.99	0.98	0.98	0.94
**isopulegol**	0.98	0.39	0.25	0.53	−0.21	0.99	0.98	0.98
**citral**	1.00	0.93	0.98	0.98	1.00	1.00	1.00	1.00
**α-phellanderene**	1.00	0.87	0.86	0.82	0.99	0.99	0.99	0.98
**linalool**	0.93	0.66	0.10	0.03	−0.29	−0.30	0.97	0.87
**carvone**	0.92	0.96	0.99	1.00	0.79	0.94	0.97	0.98

**Table 4:** logP values for selected monoterpenes. The parameter was obtained using the tool: https://biosig.lab.uq.edu.au/pkcsm/prediction (accessed on 20 December 2023).

Monoterpene	logP Value
citral	2.878
linalool	2.6698
carvone	2.4879
γ-terpinene	3.3089
α-terpinene	3.3089
α-phellandrene	3.1648
isopulegol	2.3596

## References

[1] Zielińska-Błajet M., Feder-Kubis J. (2020). Monoterpenes and Their Derivatives—Recent Development in Biological and Medical Applications. Int. J. Mol. Sci..

[2] Touhtouh J., Laghmari M., Benali T., Aanniz T., Lemhadri A., Akhazzane M., Habbadi K., Bouyahya A., Zengin G., Hammani K. (2023). Determination of the Antioxidant and Enzyme-Inhibiting Activities and Evaluation of Selected Terpenes’ ADMET Properties: In Vitro and in Silico Approaches. Biochem. Syst. Ecol..

[3] Sharifi-Rad M., Lankatillake C., Dias D.A., Docea A.O., Mahomoodally M.F., Lobine D., Chazot P.L., Kurt B., Boyunegmez Tumer T., Catarina Moreira A. (2020). Impact of Natural Compounds on Neurodegenerative Disorders: From Preclinical to Pharmacotherapeutics. J. Clin. Med..

[4] Salakhutdinov N.F., Volcho K.P., Yarovaya O.I. (2017). Monoterpenes as a renewable source of biologically active compounds. Pure Appl. Chem..

[5] Wojtunik-Kulesza K.A. (2020). Approach to Optimization of FRAP Methodology for Studies Based on Selected Monoterpenes. Molecules.

[6] Wojtunik K.A., Ciesla L.M., Waksmundzka-Hajnos M. (2014). Model Studies on the Antioxidant Activity of Common Terpenoid Constituents of Essential Oils by Means of the 2,2-Diphenyl-1-Picrylhydrazyl Method. J. Agric. Food Chem..

[7] Volcho K.P., Laev S.S., Ashraf G.M., Aliev G., Salakhutdinov N.F. (2018). Application of Monoterpenoids and their Derivatives for Treatment of Neurodegenerative Disorders. Curr Med. Chem..

[8] Wojtunik-Kulesza K.A., Targowska-Duda K., Klimek K., Ginalska G., Jóźwiak K., Waksmundzka-Hajnos M., Cieśla Ł. (2017). Volatile Terpenoids as Potential Drug Leads in Alzheimer’s Disease. Open Chem..

[9] Munteanu I.G., Apetrei C. (2021). Analytical Methods Used in Determining Antioxidant Activity: A Review. Int. J. Mol. Sci..

[10] Benzie I.F., Strain J.J. (1999). Ferric Reducing/Antioxidant Power Assay: Direct Measure of Total Antioxidant Activity of Biological Fluids and Modified Version for Simultaneous Measurement of Total Antioxidant Power and Ascorbic Acid Concentration. Methods Enzymol..

[11] Pulido R., Bravo L., Saura-Calixto F. (2000). Antioxidant Activity of Dietary Polyphenols As Determined by a Modified Ferric Reducing/Antioxidant Power Assay. J. Agric. Food Chem..

[12] Bhal S. logP—Making Sense of the Value. https://www.acdlabs.com/wp-content/uploads/download/app/physchem/making_sense.pdf.

[13] Liu F., Zhang Z., Zhang L., Meng R.N., Gao J., Jin M., Li M., Wang X.P. (2022). Effect of metal ions on Alzheimer’s disease. Brain Behav..

[14] Schober P., Boer C., Schwarte L. (2018). Correlation Coefficients: Appropriate Use and Interpretation. Anesth. Analg..

[15] de Christo Scherer M.M., Marques F.M., Figueira M.M., Peisino M.C.O., Schmitt E.F.P., Kondratyuk T.P., Endringer D.C., Scherer R., Fronza M. (2019). Wound Healing Activity of Terpinolene and α-Phellandrene by Attenuating Inflammation and Oxidative Stress in Vitro. J. Tissue Viability.

[16] Safaeian L., Sajjadi S.E., Montazeri H., Ohadi F., Javanmard S. (2020). Citral Protects Human Endothelial Cells Against Hydrogen Peroxide-Induced Oxidative Stress. Turk. J. Pharm. Sci..

[17] Bouzenna H., Hfaiedh N., Giroux-Metges M.-A., Elfeki A., Talarmin H. (2017). Biological Properties of Citral and Its Potential Protective Effects against Cytotoxicity Caused by Aspirin in the IEC-6 Cells. Biomed. Pharmacother..

[18] Henríquez C., López-Alarcón C., Gómez M., Lutz M., Speisky H. (2011). Time-Dependence of Ferric Reducing Antioxidant Power (FRAP) Index in Chilean Apples and Berries. Arch. Latinoam. Nutr..

[19] Soural I., Švestková P., Híc P., Balík J. (2022). Different Values Obtained by the FRAP Method for the Determination of Slowly and Rapidly Reacting Phenols. Acta Aliment..

